# Long COVID and its risk factors in migrants: a nationwide register study from Sweden

**DOI:** 10.1186/s12916-025-03900-x

**Published:** 2025-01-29

**Authors:** Agneta Cederström, George Frederick Mkoma, Thomas Benfield, Charles Agyemang, Marie Nørredam, Mikael Rostila

**Affiliations:** 1 Department of Public Health Sciences, Stockholm University, Stockholm, Sweden; 2 Centre for Health Equity Studies (CHESS), Stockholm University/Karolinska Institutet, Stockholm, Sweden; 3 Department of Epidemiology Research, Statens Serum Institut, Copenhagen, Denmark; 4 Danish Research Centre for Migration, Ethnicity and Health, Section of Health Services Research, Department of Public Health, University of Copenhagen, Copenhagen, Denmark; 5 Department of Infectious Diseases, Copenhagen University Hospital-Amager and Hvidovre, Hvidovre, Denmark; 6 Department of Clinical Medicine, Faculty of Health and Medical Sciences, University of Copenhagen, Copenhagen, Denmark; 7 Department of Public and Occupational Health, Amsterdam Public Health Research Institute, Amsterdam UMC, University of Amsterdam, Amsterdam, The Netherlands; 8 Division of Endocrinology, Diabetes, and Metabolism, Department of Medicine, Johns Hopkins University, Baltimore, MD USA

**Keywords:** Long COVID, Migrant health, Disease severity, Vaccination, Socioeconomic status

## Abstract

**Background:**

Many studies have found more severe COVID-19 outcomes in migrants and ethnic minorities throughout the COVID-19 pandemic, while recent evidence also suggests higher risk of longer-term consequences. We studied the risk of a long COVID diagnosis among adult residents in Sweden, dependent on country of birth and accounting for known risk factors for long COVID.

**Methods:**

We used linked Swedish administrative registers between March 1, 2020 and April 1, 2023, to estimate the risk of a long COVID diagnosis in the adult population that had a confirmed COVID-19 infection. Poisson regressions were used to calculate incidence rate ratios (IRR) of long COVID by country/region of birth. The contribution of sex, preexisting health status, disease severity, vaccination status, and socioeconomic factors to differences in long COVID diagnosis by country/region of birth were further investigated.

**Results:**

Of the 1,869,188 persons diagnosed with COVID-19 that were included, 7539 had received a long COVID diagnosis. Compared with residents born in Sweden, we found higher risks of long COVID among migrants from East Europe (IRR: 1.44 CI: 1.29–1.60), Finland (IRR: 1.36 CI: 1.15–1.61), South Asia (IRR: 1.28 CI: 1.03–1.59), Other Asia (IRR: 1.35 CI: 1.13–1.62), Other Africa (IRR: 1.48 CI: 1.17–1.87), and the Middle East (IRR: 1.43 CI: 1.27–1.63) in models adjusted for age and sex. We discovered that disease severity, i.e., whether the person was hospitalized (IRR: 18.6 CI: 17.3–20.0) or treated in an intensive care unit (IRR: 120.5 CI: 111.7–129.8), primarily contributed to the higher risk of long COVID found in migrants while the contribution of vaccinations and social conditions were moderate. Preexisting health problems did not contribute to the increased risk of long COVID in migrants.

**Conclusions:**

The greater exposure and impact of the COVID-19 virus among migrants also affected longer-term consequences. Disease severity was the most important risk factor for long COVID in migrants. The findings emphasize the need for targeted health interventions for migrant communities during an infectious disease pandemic, such as strategic vaccination campaigns and extending social insurance schemes, focusing on reducing disease severity to mitigate the longer-term health consequences of an infection.

**Supplementary Information:**

The online version contains supplementary material available at 10.1186/s12916-025-03900-x.

## Background

On May 5, 2023, the Head of the World Health Organization (WHO) declared an end to COVID-19 as a public health emergency [1]. Although the pandemic has reached an end, the lingering consequences of COVID-19 infection continues to impact millions of people worldwide [2]. Some people who have been infected with the virus that causes COVID-19 (SARS-COV-2) continues to experience longer-term health implications of previous infection, known as long COVID or post-COVID conditions (PCC) as per National Institute for Health and Care Excellence (NICE) guidelines [3]. Long COVID is broadly defined as signs, symptoms, and conditions that continue or develop after acute COVID-19 infection, which include fatigue, persistent cough, cognitive dysfunction, shortness of breath, depression, anxiety, among others. It is a multi-symptomatic disease, which have been found to cluster by severity [4, 5].

It appeared early on during the pandemic that the virus had an unequal impact on various population groups. A number of studies found higher COVID-19 infection rates, hospitalizations, and deaths in migrants and ethnic minorities throughout the pandemic in many high-income countries [6, 7] including Sweden [8, 9]. When considering that migrants and ethnic minorities were particularly affected by SARS-COV-2, they might also experience a higher risk of longer-term health complications of their infection and disease. Although empirical evidence is still scarce, some studies indicate a higher incidence of diagnosed long COVID in migrants and ethnic minorities in high-income countries such as Denmark [10], the Netherlands [11], and the USA [12], with mixed findings from the UK [13, 14]. These studies found higher risks of fatigue, cardiopulmonary symptoms (dyspnea, cough, and chest pain), and neurological symptoms (headache, depression, and memory loss) persisting beyond weeks or months after the acute phase of COVID-19 infection in migrants and ethnic minorities compared to the host populations.

Important risk factors for long COVID in general populations include sex, older age, disease severity, mechanical ventilation, comorbidities, and not receiving COVID-19 vaccine [15–20]. However, migrants have a different demographic and health profile compared to the majority population [21, 22] and it is hence unclear whether these risk factors impact the risk of long COVID also within the migrant population [23]. Identifying risk factors for long COVID in migrants are important for the prevention of longer-term consequences of virus infections in vulnerable populations in future pandemics or epidemics.

Using nationwide total-population registry data, the aim of this study was to examine the incidence rates of a long COVID diagnosis among residents in Sweden dependent on country/region of birth. Furthermore, we examined whether sex, preexisting health conditions, disease severity, social factors, and vaccinations contributed to disparities in receiving a long COVID diagnosis by country/region of birth among residents in Sweden.

## Methods

Data from multiple Swedish administrative registers was linked using a pseudonymized personal identification number. Information on age, sex, and country of birth was obtained from the Total Population Register, socioeconomic characteristics from the longitudinal integrated database for health insurance and labor market studies (LISA), positive polymerase chain reaction (PCR) tests from the electronic case reports in the notifiable infectious disease register (SmiNet), COVID-19 hospitalizations (identified by the ICD-10 code U.071) from the National Inpatient Register, and COVID-19 vaccination from the National Vaccination Register. A long COVID diagnosis was identified using the ICD-10 code U.09 in both the National Inpatient Register (hospitalizations) and National Outpatient Register (specialist primary care). In April 2021, the National Board of Health and Welfare published guidelines for health services and clinicians diagnosing long COVID in both the specialist primary care sector and in hospitals [24].

### Variables

Country/region of birth was categorized into Sweden, East Europe, Finland, Horn of Africa (Somalia, Ethiopia, Eritrea), Middle East, North America, Other Nordic (Norway, Denmark, Iceland, Faroe Islands), Other Africa, Other Asia, South America, South Asia (India, Sri Lanka, Pakistan, Afghanistan), and Western Europe. In addition, a more detailed country/region of birth category was used with 50 groups to see which countries drove the findings on the more coarse-grained categorization. Age was grouped into 0–24, 25–29, 30–34, 35–39, 40–44, 45–49, 50–54, 55–59, 60–64, 65–69, 70–74, 75–79, 80–84, 85–120. Education was derived from ISCED-11 and was classified into primary, secondary, post-secondary, and unknown. Individual disposable income was divided into quartiles (Q1—lowest, Q2, Q3, Q4—highest). Broad skill level was derived from ISCO-08 (International Standard Classification of Occupations) and is categorized as the following, 3,4: managers, professionals, technicians, and associate professionals; 2: clerical, service, and sales workers, skilled agricultural, forestry and fishery worker, craft and related trade workers, and plant and machine operators, and assemblers; 1: elementary occupations; AF: armed forces; and X: not elsewhere classified [25]. A Charlson comorbidity index (CCI) score was calculated as an indicator of underlying health and categorized into 0, 1, and 2 + . Disease severity was categorized by whether it was a “confirmed case,” the patient was “hospitalized,” or in need of “intensive care.” Vaccination status was based on whether the individual had received no vaccinations, “unvaccinated,” or had received vaccination *after* the positive PCR test, “post-infection vaccine.” In the cases where vaccinations had been administered *before* the positive test, we also added the number of doses received prior to the positive test such as “pre-infection vaccine 1 dose,” “pre-infection vaccine 2 dose,” and “pre-infection vaccine 3 dose.”

### Study population

The study population is defined by all individuals alive and 18 years of age and older, and were resident in Sweden at the end of 2019, who tested positive for the SARS-COV-2 virus before July 1, 2022. There were 18 instances of duplicated identification numbers and 6813 individuals without socioeconomic data which were removed.

### Method

Using Poisson regression analyses, we estimated the risk of getting a long COVID diagnosis dependent on country/region of birth. In the analyses, follow-up was defined from March 1, 2020 until first diagnosis with long COVID, death, emigration, or end of follow-up on April 1, 2023, whichever occurred first. The log of the follow-up was included as an offset in the models and incidence rate ratios (IRR) were calculated with a 95% confidence interval (CI). Initially an unadjusted model was run (M0), and then we adjusted for age and sex (M1). Then, we additionally adjusted for underlying health as measured by a categorized CCI (M2), disease severity (M3), vaccination status (M4), and socioeconomic status as indicated by income, education, and occupational skill level (M5). A last model was run which included *all* factors: age, sex, CCI, disease severity, vaccination status, income, education, and occupational skill level (M6). In order to investigate whether the relationship between region/country of birth and risk of a long COVID diagnosis was modified by the explanatory factors, additional interaction models were examined and a least likelihood ratio test conducted to assess the statistical significance of the interaction. As the variants of the virus (alpha, delta, omicron, etc.) is another possible contributing factor, we also ran models where the timing of the infection was taken into account, as a proxy for the variant of the virus. The pandemic was loosely defined by three waves (wave 1: 2020–03-01–2020–09-01, wave 2: 2020–09-02–2021–07-01, wave 3: 2021–07-02–2023–04-01), and we investigated how the risk of receiving a long COVID diagnosis evolved over these three waves, and how the relative risks by country of birth differed by wave. Since the coverage of testing for COVID-19 in Sweden was not very extensive especially in the first wave and there is a selection effect in testing behavior, additional sensitivity analyses were run on the full population. Data management and statistical analyses were performed using R 4.3.0 (R Core Team, 2023; R Foundation for Statistical Computing, Vienna, Austria).

## Results

Between Dec 2019 and July 2022, 1,869,188 adult individuals tested positive for COVID-19, of which 7539 had a long COVID diagnosis. In the total population of 7,992,298 individuals, there were 9809 individuals with a long COVID diagnosis.

Table 1 shows the study population characteristics for those with a confirmed infection, the total population, and for those with a confirmed infection who had received a long COVID diagnosis. We observe some of the selection effects into testing behavior where women with higher income, longer education, and occupations that require longer schooling are more prone to getting tested and hence also of having a positive test. This despite the knowledge that men with lower incomes and education had a higher risk of serious consequences of a COVID-19 infection, and most likely also were more exposed to the virus.

**Table 1:** Descriptive characteristics of study population (*N* = 1,869,188) with confirmed COVID-19 infection, the total adult population (*N* = 7,992,298), and those with a long COVID diagnosis (*N* = 7539)

	**Confirmed cases**		**Total population**		**Long COVID diagnosis**	
	Mean	St.Dev	Mean	St.Dev	Mean	St.Dev
**Age**	43.6	16.3	50.5	19.2	54.4	15.7
**Sex**	*N*	%	*N*	%	*N*	%
Female	999,875	53.5	3,999,272	50.0	3973	52.7
Male	869,313	46.5	3,993,011	50.0	3566	47.3
**Region of origin**	*N*	%	*N*	%	*N*	%
East Europe	93,984	5.0	368,143	4.6	520	6.9
Finland	20,377	1.1	141,903	1.8	175	2.3
Horn of Africa	26,235	1.4	111,849	1.4	77	1.0
Middle East	121,019	6.5	394,161	4.9	560	7.4
North America	4604	0.2	17,446	0.2	18	0.2
Other Africa	17,069	0.9	76,045	1.0	82	1.1
Other Asia	32,313	1.7	145,028	1.8	137	1.8
Other Nordic	14,428	0.8	77,339	1.0	64	0.8
South America	20,149	1.1	70,378	0.9	123	1.6
South Asia	28,388	1.5	108,809	1.4	90	1.2
Sweden	1,462,920	78.3	6,339,802	79.3	5577	74.0
West Europe	27,702	1.5	141,380	1.8	116	1.5
**Charlson comorbidity index**	*N*	%	*N*	%	*N*	%
0	1,783,318	98.8	7,846,455	98.2	7098	94.2
1	10,084	0.5	66,282	0.8	202	2.8
2 +	12,654	0.7	79,546	1.0	239	3.2
**Disease severity**	*N*	%	*N*	%	*N*	%
Confirmed infection	1,783,318	95.4	1,754,720	22.0	3515	46.6
Hospitalization	77,316	4.1	105,914	1.3	2470	32.8
ICU	8554	0.5	8554	0.1	1554	20.6
**Vaccine status**	*N*	%	*N*	%	*N*	%
Unvaccinated	228,553	12.2	1,035,405	12.9	1107	14.7
Post-infection vaccine	775,452	41.5	–	–	5116	67.8
Pre-infection vaccine 1 dose	57,587	3.1	–	–	293	3.9
Pre-infection vaccine 2 dose	535,403	28.6	–	–	591	7.8
Pre-infection vaccine 3 dose	272,192	14.6	–	–	432	5.7
**Disposable income**	*N*	%	*N*	%	*N*	%
0–24	344,019	18.4	2,064,257	25.8	1688	22.4
25–49	428,647	22.9	2,025,882	25.3	1806	24.0
50–74	524,000	28.0	1,977,993	24.7	2073	27.5
75–100	572,522	30.6	1,924,151	24.1	1972	26.2
**Education**	*N*	%	*N*	%	*N*	%
Post-secondary	774,943	41.5	2,955,950	37.0	2932	38.9
Secondary	819,520	43.8	3,457,489	43.3	3194	42.4
Primary	242,754	13.0	1,384,320	17.3	1304	17.3
Unknown	31,971	1.7	194,524	2.4	109	1.4
**Occupational skill level**^a^	*N*	%	*N*	%	*N*	%
3,4	664,092	34.5	2,223,564	27.8	2378	31.5
2	725,329	38.8	2,498,067	31.2	2380	31.6
1	75,830	4.1	310,485	3.9	264	3.5
AF	3204	0.2	11,414	0.1	–	0.1
X	420,733	22.5	2,948,753	36.9	2513	33.3

^a^3,4: managers, professionals, technicians, and associate professionals; 2: clerical, service, and sales workers, skilled agricultural, forestry and fishery worker, craft and related trade workers, and plant and machine operators, and assemblers; 1: elementary occupations; AF: armed forces; X: not elsewhere classified

Table 2 shows the incidence rate ratios with 95% confidence intervals estimated with Poisson regressions. Most migrant groups have an elevated risk of long COVID such as migrants from East Europe (M1 IRR: 1.44 CI: 1.29–1.60), Finland (M1 IRR: 1.36 CI: 1.15–1.61), South Asia (M1 IRR: 1.28 CI: 1.03–1.59), Other Asia (M1 IRR: 1.35 CI: 1.13–1.62), Other Africa (M1 IRR: 1.48 CI: 1.17–1.87), and the Middle East (M1 IRR: 1.34 CI: 1.20–1.48) when compared to residents born in Sweden. While underlying health barely changes the estimates, and there was some attenuation in the estimates for vaccination and socioeconomic status, the estimates were drastically reduced when adjusting for disease severity. The incidence rate ratios were actually lower for migrants from Horn of Africa (M3 IRR: 0.55 CI: 0.44–0.68), the Middle East (M3 IRR: 0.76 CI: 0.69–0.83), and Other Asia (M3 IRR: 0.73 CI: 0.57–0.92), when compared to residents born in Sweden, and had completely attenuated for migrants from East Europe (M3 IRR: 0.96 CI: 0.87–1.05), Finland (M3 IRR: 0.95 CI: 0.81–1.10), and Other Africa (M3 IRR: 0.84 CI: 0.67–1.05) after adjusting for disease severity. The drastic effect of disease severity could also be seen in the risk of a long COVID diagnosis elevated by more than a 100-fold (IRR: 120.4 CI: 111.7–129.8) after being treated at the intensive care unit following a COVID-19 infection, while being hospitalized with a COVID-19 infection increases the risk of a long COVID diagnosis about 20-fold (IRR: 18.6 CI: 17.3–19.9). Similarly, the sensitivity analysis for the total population which is shown in Additional file 1: Table S1 we see elevated IRR for migrants from Eastern Europe, the Middle East, and South America which are drastically reduced when accounting for disease severity.

**Table 2:** Incidence rate ratios (IRR) with 95% confidence intervals (95%CI) estimated with Poisson regression. The Akaike information criteria (AIC) is reported as measure of the goodness-of-fit for each model

	**M0** AIC: 56284	**M1** AIC: 52473	**M2** AIC: 52187	**M3** AIC: 36788	**M4** AIC: 49128	**M5** AIC: 52317	**M6** AIC: 35292
	IRR (95%C)	IRR (95%CI)	IRR (95%CI)	IRR (95%CI)	IRR (95%CI)	IRR (95%CI)	IRR (95%CI)
**Country/region of origin**							
Sweden	1 (ref)	1 (ref)	1 (ref)	1 (ref)	1 (ref)	1 (ref)	1 (ref)
East Europe	1.45 (1.30–1.61)	1.44 (1.29–1.60)	1.43 (1.29–1.59)	0.96 (0.87–1.05)	1.16 (1.05–1.29)	1.36 (1.22–1.51)	0.92 (0.83–1.01)
Finland	2.33 (1.97–2.75)	1.36 (1.15–1.61)	1.34 (1.13–1.58)	0.95 (0.81–1.10)	1.29 (1.09–1.53)	1.31 (1.11–1.55)	0.93 (0.80–1.08)
Horn of Africa	0.77 (0.61–0.97)	1.04 (0.82–1.31)	1.03 (0.82–1.30)	0.55 (0.44–0.68)	0.88 (0.70–1.11)	0.93 (0.74–1.17)	0.54 (0.43–0.68)
Middle East	1.21 (1.09–1.34)	1.34 (1.20–1.48)	1.33 (1.20–1.48)	0.76 (0.69–0.83)	1.06 (0.95–1.17)	1.18 (1.06–1.31)	0.71 (0.65–0.78)
North America	1.02 (0.64–1.62)	1.13 (0.71–1.80)	1.13 (0.71–1.80)	0.86 (0.55–1.34)	1.03 (0.65–1.64)	1.08 (0.68–1.72)	0.83 (0.54–1.30)
Other Africa	1.25 (0.99–1.58)	1.48 (1.17–1.87)	1.47 (1.17–1.85)	0.84 (0.67–1.05)	1.24 (0.99–1.57)	1.37 (1.09–1.73)	0.79 (0.63–0.99)
Other Asia	1.10 (0.92–1.32)	1.35 (1.13–1.62)	1.35 (1.12–1.62)	0.83 (0.70–0.99)	1.27 (1.06–1.52)	1.26 (1.05–1.51)	0.81 (0.68–0.97)
Other Nordic	1.17 (0.91–1.51)	0.98 (0.76–1.26)	0.97 (0.76–1.25)	0.96 (0.75–1.23)	0.96 (0.75–1.24)	0.94 (0.73–1.21)	0.98 (0.77–1.26)
South America	1.60 (1.32–1.93)	1.53 (1.27–1.85)	1.54 (1.27–1.86)	1.02 (0.85–1.23)	1.37 (1.14–1.66)	1.46 (1.21–1.77)	0.96 (0.80–1.14)
South Asia	0.83 (0.66–1.04)	1.28 (1.03–1.59)	1.28 (1.03–1.59)	0.77 (0.62–0.95)	1.13 (0.91–1.41)	1.16 (0.94–1.45)	0.75 (0.61–0.93)
West Europe	1.10 (0.91–1.33)	1.17 (0.97–1.42)	1.17 (0.97–1.42)	1.08 (0.89–1.30)	1.08 (0.90–1.31)	1.14 (0.94–1.38)	1.04 (0.87–1.26)
**Sex**							
Female		1 (ref)	1 (ref)	1 (ref)	1 (ref)	1 (ref)	1 (ref)
Male		1.01 (0.93–1.10)	1.00 (0.92–1.09)	0.75 (0.71–0.80)	0.96 (0.88–1.05)	1.02 (0.94–1.11)	0.74 (0.70–0.78)
**Age category**							
(18,25]		1 (ref)	1 (ref)	1 (ref)	1 (ref)	1 (ref)	1 (ref)
(25,30]		2.12 (1.68–2.67)	2.12 (1.68–2.66)	1.95 (1.56–2.43)	2.24 (1.83–2.75)	2.12 (1.69–2.67)	1.86 (1.55–2.24)
(30,35]		2.15 (1.72–2.68)	2.15 (1.72–2.68)	1.97 (1.59–2.43)	2.40 (1.97–2.93)	2.25 (1.80–2.81)	1.91 (1.59–2.30)
(35,40]		3.32 (2.65–4.17)	3.31 (2.64–4.16)	3.05 (2.46–3.79)	3.87 (3.17–4.71)	3.61 (2.88–4.53)	3.03 (2.53–3.62)
(40,45]		4.17 (3.27–5.33)	4.16 (3.26–5.31)	3.58 (2.83–4.53)	4.78 (3.91–5.84)	4.63 (3.64–5.90)	3.50 (2.93–4.20)
(45,50]		5.70 (4.57–7.10)	5.65 (4.54–7.04)	4.17 (3.41–5.11)	6.34 (5.18–7.75)	6.37 (5.14–7.90)	4.00 (3.40–4.71)
(50,55]		6.62 (5.33–8.22)	6.54 (5.27–8.11)	4.16 (3.43–5.03)	7.25 (5.86–8.95)	7.34 (5.94–9.08)	3.98 (3.39–4.68)
(55,60]		8.48 (6.82–10.55)	8.30 (6.68–10.31)	4.38 (3.64–5.28)	9.33 (7.51–11.61)	9.21 (7.44–11.41)	4.24 (3.61–4.98)
(60,65]		10.52 (8.38–13.19)	10.16 (8.12–12.72)	3.97 (3.28–4.79)	11.25 (8.97–14.13)	11.09 (8.90–13.82)	3.87 (3.28–4.56)
(65,70]		13.61 (10.85–17.07)	12.78 (10.21–16.01)	3.36 (2.76–4.08)	14.95 (11.97–18.68)	13.31 (10.65–16.63)	3.48 (2.92–4.15)
(70,75]		16.17 (12.81–20.41)	14.64 (11.62–18.43)	2.96 (2.43–3.61)	18.86 (15.01–23.69)	13.87 (10.99–17.52)	3.36 (2.81–4.03)
(75,80]		18.31 (14.32–23.40)	15.88 (12.43–20.28)	2.76 (2.24–3.39)	22.03 (17.50–27.74)	14.86 (11.57–19.07)	3.29 (2.72–3.97)
(80,85]		13.92 (10.58–18.33)	11.78 (8.99–15.43)	2.19 (1.74–2.75)	17.38 (13.53–22.34)	10.88 (8.21–14.42)	2.70 (2.19–3.34)
(85,120]		8.06 (5.85–11.10)	6.92 (5.10–9.40)	1.55 (1.20–1.99)	10.26 (7.67–13.71)	6.06 (4.34–8.47)	1.95 (1.53–2.50)
**Charlson index**							
0			1 (ref)				1 (ref)
1			2.67 (2.27–3.16)				1.49 (1.28–1.72)
2 +			2.60 (2.24–3.02)				1.26 (1.10–1.44)
**C19 disease severity**							
Confirmed case				1 (ref)			1 (ref)
Hospitalized				18.56 (17.3–19.9)			14.8 (13.9–15.8)
Intensive care				120.4 (111.7–129.8)			89.9 (83.8–96.7)
**Vaccine status**							
No vaccine					1 (ref)		1 (ref)
Post-infection vaccine					1.02 (0.93–1.10)		1.17 (1.09–1.26)
Pre-infection vaccine 1					0.70 (0.61–0.82)		1.00 (0.87–1.14)
Pre-infection vaccine 2					0.22 (0.19–0.25)		0.37 (0.33–0.41)
Pre-infection vaccine 3					0.17 (0.15–0.19)		0.33 (0.30–0.38)
**Disposable income quartiles**							
Q1						1 (ref)	1 (ref)
Q2						0.87 (0.78–0.97)	1.02 (0.94–1.10)
Q3						0.81 (0.73–0.90)	1.03 (0.96–1.12)
Q4						0.69 (0.61–0.78)	0.93 (0.86–1.02)
**Education**							
Primary						1 (ref)	1 (ref)
Secondary						0.92 (0.83–1.02)	1.05 (0.97–1.12)
Post-secondary						0.92 (0.83–1.02)	1.18 (1.09–1.28)
Unknown						0.81 (0.65–1.00)	0.85 (0.69–1.05)
**Occupational skill level**^a^							
3,4						1 (ref)	1 (ref)
2						0.93 (0.84–1.04)	0.95 (0.88–1.01)
1						0.92 (0.79–1.07)	0.82 (0.72–0.94)
AF						0.54 (0.20–1.42)	0.55 (0.21–1.45)
X						1.16 (1.04–1.29)	0.86 (0.79–0.93)

^a^3,4: managers, professionals, technicians, and associate professionals; 2: clerical, service, and sales workers, skilled agricultural, forestry and fishery worker, craft and related trade workers, and plant and machine operators, and assemblers; 1: elementary occupations; AF: armed forces; X: not elsewhere classified

Although the effect of the other explanatory factors (underlying health, vaccinations, and socioeconomic status) only moderately attenuated the IRR for the migrant groups, they were nonetheless important predictors for a long COVID diagnosis. Our results show that being vaccinated before the infection reduced the risk of a long COVID diagnosis, and that each additional dose further reducing this risk (M4 post-infection vaccine: dose 1 IRR: 0.70 CI: 0.61–0.82, dose 2 IRR: 0.22 CI: 0.19–0.25, dose 3 IRR: 0.17 CI: 0.15–0.19). In addition, we observed a socioeconomic gradient where individuals with high income (M4 Q4 IRR: 0.69 CI: 0.61–0.78 compared to Q1) had lower risks for a long COVID diagnosis, and those with underlying health problems also showed higher risks (M2 CCI 2 + IRR: 2.60 CI: 2.24–3.02 compared to a CCI of 0). No clear pattern of association was observed for occupational skill level. There was no observed difference between the sexes except after adjustment for disease severity, where men have a lower risk (M4 IRR: 0.75 CI: 0.71–0.80) than women.

The results for the seven largest migrant groups by country of birth are shown in Table 2 with the rest of the countries detailed in Additional file 1: Table S2. Migrants from Iran (M1 IRR: 1.27 CI: 1.09–1.49), Iraq (M1 IRR: 1.29 CI: 1.05–1.57), Syria (M1 IRR: 1.31 CI: 1.10–1.55), and Yugoslavia (M1 IRR: 1.83 CI: 1.54–2.16) have elevated risks, which completely attenuated or even reversed as in the case for Afghanistan (M3 IRR: 0.66 CI: 0.49–0.89), Iran (M3 IRR: 0.69 CI: 0.60–0.80), Iraq (M3 IRR: 0.87 CI: 0.72–1.06), and Somalia (M3 IRR: 0.44 CI: 0.30–0.64) after adjusting for disease severity. We observed that across the migrant groups from the Horn of Africa (Ethiopia, Eritrea, and Somalia), South Asia (Afghanistan, Pakistan, India, and Sri Lanka), and the Middle East (Syria, Lebanon, Iraq, Iran, Turkey) the patterns were similar across countries within the region of origin with the results not driven by any particular country. Much more heterogeneity was observed across the other migrant groups from Other Asia, East Europe, and South America where, for example, South Vietnam or Yugoslavia have quite high risks and might be driving the association on the regional group level (Table 3).

**Table 3:** Incidence rate ratios (IRR) with 95% confidence intervals (95%CI) estimated with Poisson regression with long COVID diagnosis by country of origin

	**M0**	**M1**	**M2**	**M3**	**M4**	**M5**	**M6**
**Country/region of origin**							
Sweden	1 (ref)s	1 (ref)	1 (ref)	1 (ref)	1 (ref)	1 (ref)	1 (ref)
Afghanistan	0.67 (0.48–0.93)	1.19 (0.88–1.62)	1.19 (0.87–1.62)	0.66 (0.49–0.89)	1.00 (0.74–1.37)	1.04 (0.76–1.41)	0.66 (0.49–0.89)
Finland	2.33 (1.97–2.75)	1.37 (1.15–1.62)	1.34 (1.14–1.59)	0.95 (0.81–1.11)	1.29 (1.09–1.53)	1.32 (1.11–1.56)	0.93 (0.80–1.09)
Iran	1.38 (1.13–1.69)	1.27 (1.09–1.49)	1.27 (1.09–1.49)	0.69 (0.60–0.80)	0.99 (0.85–1.16)	1.14 (0.97–1.33)	0.64 (0.55–0.74)
Iraq	1.15 (0.98–1.35)	1.29 (1.05–1.57)	1.29 (1.05–1.57)	0.87 (0.72–1.06)	1.13 (0.92–1.38)	1.21 (0.99–1.47)	0.80 (0.66–0.97)
Somalia	0.66 (0.45–0.96)	0.98 (0.68–1.43)	0.97 (0.67–1.42)	0.44 (0.30–0.64)	0.82 (0.56–1.19)	0.86 (0.59–1.26)	0.44 (0.30–0.64)
Syria	1.05 (0.88–1.25)	1.31 (1.10–1.55)	1.30 (1.09–1.54)	0.72 (0.62–0.85)	1.00 (0.84–1.19)	1.09 (0.91–1.29)	0.69 (0.59–0.81)
Yugoslavia	2.23 (1.88–2.64)	1.83 (1.54–2.16)	1.81 (1.53–2.14)	1.12 (0.96–1.31)	1.47 (1.24–1.74)	1.73 (1.46–2.04)	1.08 (0.92–1.26)

M0: unadjusted; M1: adjusted for age and sex; M2: adjusted for age, sex, and Charlson comorbidity index (CCI); M3: adjusted for age, sex, and COVID-19 disease severity; M4: adjusted for age, sex, and vaccination status; M5: adjusted for age, sex, and socioeconomic factors (disposable income, education, and occupational skill level); M6: adjusted for age, sex, CCI, disease severity, vaccination status, and socioeconomic factors

Additional file 1: Table S3 shows the results when considering the timing of the infections where we found that the risk of a long COVID diagnosis did decrease across the three waves even after accounting for vaccinations. However, this did not contribute significantly to the disparities across country/region of birth compared with residents born in Sweden, except for residents born in the Middle East where the excess risk disappears altogether, and it is no longer statistically significant for South Asia. The within wave relative risks across country/region of birth detailed in Additional file 1: Table S4 show some interesting modifications, where, for example, it increases across the waves for residents born in Finland, stays stable for those born in East Europe, and decreases across waves for those born in Other Africa, Other Asia, and South America. In all models which adjust for disease severity, the excess risks seen in the migrant groups disappear or reverse.

In the interaction models, the least-likelihood ratio tests all had *p* < 0.05. Thus, the IRR by country/region of birth was modified by the explanatory variables. A summary of these results is shown in Additional file 1: Figs. S1–S6. In the interaction analysis with sex (Additional file 1: Fig. S1), we see that males had larger disparities than females and in more groups. Thus, the disparities seen by country of birth were largely driven by the male population. In the case of underlying health (Additional file 1: Fig. S2), we observed the effect of having a CCI score of 1 and 2 or more, to be a stronger predictor for a long COVID diagnosis for migrants from Horn of Africa, Middle East, and South America, compared to residents born in Sweden signaling higher vulnerability with worse health. The observed reverse effect for disease severity (Additional file 1: Fig. S3) where migrants from Horn of Africa, Middle East, and Other Africa actually had lower relative risks for a long COVID diagnosis per higher infection severity compared to the Swedish born, signaling lower vulnerability for these migrant groups when needing hospitalization or ICU. When looking at the interaction effects for income and education (Additional file 1: Figs. S5–S6), we found a clear gradient for the Swedish born, which was not as apparent for many of the migrant groups meaning that having lower income or education was not as strong a predictor of a long COVID diagnosis within these migrant groups when compared with the Swedish born group.

## Discussion

This study examined the risk of a long COVID diagnosis among residents in Sweden by country of birth and to what extent various risk factors such as sex, preexisting health conditions, disease severity, social inequalities, and vaccinations contributed to this association. Compared to residents born in Sweden, many migrant groups faced higher risks of a long COVID diagnosis, with inferences strongest for migrants from East Europe, Finland, South Asia, Other Asia, Other Africa, and the Middle East. These findings are in line with previous evidence from Denmark and the Netherlands, showing higher risks of long COVID diagnosis in migrant and ethnic minorities. The results highlight that migrant groups were not only more significantly impacted by the virus in the shorter-term during the pandemic but also, to a greater extent, suffer from its longer-term consequences.

We further examined whether previously suggested risk factors for long COVID in general populations contributed to the association between country of birth and long COVID diagnosis. We found that individuals hospitalized or treated in an intensive care unit had a large risk of being diagnosed with long COVID, which is in line with previous studies. The results further suggest that much of the increased risks of long COVID diagnosis in migrants is explained by disease severity, i.e., being hospitalized or treated at an intensive care unit due to COVID-19 infection. These findings align with previous studies that have found higher risks of ICU and death due SARS-COV-2 infection in migrants throughout the pandemic. The higher rates of ICU care in migrant and ethnic minorities can be understood in light of higher exposure to the virus in earlier stages of the pandemic in which socioeconomic and living conditions contributed to higher contagion among these groups at a time with less developed treatment options [26–28], perhaps in conjunction with disparities in health care access and health care seeking behavior. Lower rates of vaccination in migrants and poorer socioeconomic conditions (education, income, and occupational skill level) did mediate some of the association between country of birth and long COVID while underlying health problems did not contribute to this association. Although the mediating effect of socioeconomic status and vaccinations was lower, the results nonetheless confirmed some previously published results on the direction and strength of the associations, especially in regards to the protective effects of the vaccine (administered before the infection) against a long COVID diagnosis [29]. Thus, disease severity is the most important risk factor of long COVID diagnosis in migrants but lower vaccination rates and poorer socioeconomic conditions also contribute to some extent.

We also found that the incidence rates were modified by these risk factors, thus differentially impacting the migrant groups risk of a long COVID diagnosis compared to the Swedish born population. Sometimes the impact was higher or lower depending on group and explanatory variable. For example, male residents who were not born in Sweden had higher relative risks across country/region of birth compared to female residents across most migrant groups, while disparities by disease severity was lower for some migrant group compared to residents born in Sweden.

### Strengths and limitations

The use of total population data, longitudinal follow-up of diagnosed long COVID cases, reliable information on vaccinations, socio-demographic conditions, underlying health problems, and hospitalizations/ICU admission from registers should be considered important strengths of the study. A limitation of the study is the fact that testing capacity and frequency varied throughout the pandemic in Sweden due to changes in governmental guidelines and routines concerning testing in the health care system [30]. For instance, the testing capacity was very limited during the first wave of the pandemic (spring 2020) in Sweden, while in the last major wave (omicron variant Dec 2022–Feb 2023) many infections were not confirmed with a PCR test. Therefore, many positive COVID-19 cases might not be recorded in the register data used and hence we might underestimate the population at risk for long COVID, especially those infected during the first and last major waves. Also, much of the testing done in Sweden was initiated by the individuals themselves so there is a socioeconomic bias towards those that have better health literacy and seek health care more generally. The limitations in testing may result in an underestimation of our IRR, as it is likely that migrants, who were harder hit, were tested less frequently. We performed sensitivity analysis on the total population, including both confirmed cases and cases with no confirmed infection. The results were fairly similar when compared to the main findings. Furthermore, there is a risk that many individuals with signs and symptoms of long COVID did not receive a long COVID diagnosis since a diagnosis could depend on factors such as health care demand, language proficiency, and health literacy. Hence, we might underestimate the incidence of long COVID. This might particularly be a problem in the migrant population when considering their lower health care demands, health literacy, and language proficiency as compared with residents born in Sweden [31]. Finally, hospitalization does not necessarily reflect disease severity and could depend on factors such as availability and demand of treatment. Also, there is a small note of caution in over-interpreting the results of the number of vaccinations, where the timing of the variants may be a confounder since the omicron wave (which was a much less virulent strain) was after many people had taken their second dose.

## Conclusions

We found that the greater exposure and impact of the COVID-19 virus among migrants during the pandemic also affected the longer-term consequences of infection in this at-risk population in Sweden. Disease severity was the most important risk factor of long COVID in migrants. In a future virus pandemic, public health measures such as targeted vaccination campaigns, extended social insurance schemes [32], and rehousing at-risk individuals in multigenerational households, aimed at limiting the contagion in vulnerable social groups such as migrant and ethnic minority communities would also benefit their long-term health consequences.

## Supplementary Information

Additional file 1. Results from sensitivity analysis for the total adult population (Table S1), results for the complete list of all countries available in data set (Table S2), and results for the analyses for three waves of the pandemic (Table S3). It also includes figures from the interaction analyses showing the predicted counts (estimated marginal means) for interactions between region of origin and sex (Fig. S1), underlying health (Fig. S2), infection severity (Fig. S3), vaccination (Fig. S4), disposable income (Fig. S5), and education (Fig. S6).

## Data Availability

The data that support the findings of this study are available from National Board of health and Welfare https://bestalladata.socialstyrelsen.se/bestalla-microdata-for-statistikandamal/ and Statistics Sweden https://www.scb.se/vara-tjanster/bestall-data-och-statistik/ but are not publicly available due to the Public Access to Information and Secrecy Act (OSL) which protects sensitive personal data. Data access is however available from the authors upon reasonable request and with permission of the Ethical Review Agency https://etikprovningsmyndigheten.se/.

## Abbreviations

IRR: Incidence rate ratios
CI: Confidence interval
CCI: Charlson comorbidity index
