# Supplementary material for: Long COVID and its risk factors in migrants: a nationwide register study from Sweden
Source: BMC Med. 2025 Jan 29;23:53. doi: 10.1186/s12916-025-03900-x (PMC11776292; doi:10.1186/s12916-025-03900-x)
Supplement: Supplementary file 1 — Additional file 1. Results from sensitivity analysis for the total adult population (Table S1), results for the complete list of all countries available in data set (Table S2), and results for the analyses for three waves of the pandemic (Table S3). It also includes figures from the interaction analyses showing the predicted counts (estimated marginal means) for interactions between region of origin and sex (Fig. S1), underlying health (Fig. S2), infection severity (Fig. S3), vaccination (Fig. S4), disposable income (Fig. S5), and education (Fig. S6). [file 12916_2025_3900_MOESM1_ESM.docx]

**Table S1:** Incidence rate ratios (IRR) with 95% confidence intervals (95%CI) estimated with Poisson regression with long COVID diagnosis as outcome and region of origin as exposure for the adult population. Results are for an unadjusted model (M0), an age and sex adjusted model (M1), and additionally adjusted for each of the mediating variables: underlying health (M2), disease severity (M3), vaccination status (M4), socioeconomic status (M5), and a last model which include all the mediating variables (M6). The estimates for the covariate and mediating variables are not shown in table but available upon request.

|  | **M0** | **M1** | **M2** | **M3** | **M4** | **M5** | **M6** |
| --- | --- | --- | --- | --- | --- | --- | --- |
|  | IRR(95%CI) | IRR(95%CI) | IRR(95%CI) | IRR(95%CI) | IRR(95%CI) | IRR(95%CI) | IRR(95%CI) |
| **Country/Region of origin** | | | | | | | |
| Sweden | 1(ref) | 1(ref) | 1(ref) | 1(ref) | 1(ref) | 1(ref) | 1(ref) |
| East Europe | 1.39 (1.25-1.55) | 1.34 (1.21-1.50) | 1.34 (1.20-1.49) | 0.96 (0.87-1.05) | 1.23 (1.11-1.37) | 1.42 (1.28-1.58) | 0.98 (0.89-1.08) |
| Finland | 1.30 (1.09-1.56) | 1.17 (0.97-1.41) | 1.16 (0.97-1.38) | 0.95 (0.81-1.11) | 1.15 (0.95-1.38) | 1.18 (0.98-1.42) | 0.95 (0.81-1.11) |
| Horn of Africa | 0.63 (0.50-0.79) | 0.73 (0.58-0.91) | 0.73 (0.58-0.91) | 0.55 (0.44-0.68) | 0.69 (0.55-0.87) | 0.78 (0.62-0.99) | 0.59 (0.47-0.74) |
| Middle East | 1.35 (1.21-1.50) | 1.39 (1.25-1.56) | 1.39 (1.25-1.55) | 0.76 (0.69-0.83) | 1.33 (1.20-1.48) | 1.45 (1.30-1.61) | 0.80 (0.73-0.88) |
| North America | 1.10 (0.73-1.67) | 1.06 (0.70-1.61) | 1.07 (0.70-1.62) | 0.86 (0.55-1.34) | 1.02 (0.67-1.55) | 1.09 (0.72-1.65) | 0.87 (0.56-1.36) |
| Other Africa | 1.10 (0.88-1.36) | 1.12 (0.90-1.39) | 1.12 (0.90-1.39) | 0.84 (0.67-1.05) | 1.05 (0.85-1.31) | 1.18 (0.95-1.46) | 0.85 (0.68-1.07) |
| Other Asia | 0.88 (0.74-1.05) | 0.86 (0.72-1.03) | 0.87 (0.72-1.03) | 0.83 (0.70-0.99) | 0.84 (0.70-1.00) | 0.92 (0.77-1.10) | 0.87 (0.73-1.04) |
| Other Nordic | 0.84 (0.66-1.06) | 0.79 (0.62-1.01) | 0.79 (0.62-1.00) | 0.96 (0.75-1.23) | 0.77 (0.60-0.97) | 0.83 (0.65-1.05) | 0.98 (0.76-1.25) |
| South America | 1.73 (1.45-2.07) | 1.62 (1.35-1.94) | 1.62 (1.36-1.94) | 1.02 (0.85-1.23) | 1.57 (1.31-1.87) | 1.67 (1.40-1.99) | 1.04 (0.86-1.25) |
| South Asia | 0.78 (0.62-0.98) | 1.01 (0.81-1.25) | 1.01 (0.81-1.25) | 0.77 (0.62-0.95) | 0.99 (0.80-1.23) | 1.08 (0.87-1.33) | 0.83 (0.67-1.03) |
| West Europe | 0.87 (0.73-1.05) | 0.90 (0.76-1.08) | 0.91 (0.76-1.08) | 1.08 (0.89-1.30) | 0.84 (0.71-1.01) | 0.94 (0.78-1.12) | 1.07 (0.89-1.29) |

**Table S2**: Incidence rate ratios (IRR) with 95% confidence intervals (95%CI) estimated with Poisson regression with long COVID diagnosis as outcome and country of birth as exposure for the positive PCR test study population. Results are for an age and sex adjusted model (M1), and additionally adjusted for each of the mediating variables: underlying health (M2), disease severity (M3), vaccination status (M4), socioeconomic status (M5), and a last model which include all the mediating variables (M6). The estimates for the covariate and mediating variables are not shown in table but available upon request.

|  | **M1** | **M2** | **M3** | **M4** | **M5** | **M6** |
| --- | --- | --- | --- | --- | --- | --- |
| **Country/region of origin** | | | | | | |
| Sweden | 1(ref) | 1(ref) | 1(ref) | 1(ref) | 1(ref) | 1(ref) |
| Afghanistan | 1.19 (0.88-1.62) | 1.19 (0.87-1.62) | 0.66 (0.49-0.89) | 1.00 (0.74-1.37) | 1.04 (0.76-1.41) | 0.66 (0.49-0.89) |
| Austria | 1.44 (0.64-3.23) | 1.42 (0.64-3.19) | 1.13 (0.50-2.55) | 1.39 (0.62-3.11) | 1.43 (0.64-3.20) | 1.12 (0.49-2.53) |
| Brazil | 0.70 (0.29-1.68) | 0.70 (0.29-1.67) | 0.66 (0.28-1.56) | 0.68 (0.29-1.64) | 0.67 (0.28-1.60) | 0.65 (0.28-1.53) |
| Chile | 1.50 (1.14-1.96) | 1.50 (1.14-1.96) | 1.00 (0.77-1.30) | 1.32 (1.00-1.73) | 1.43 (1.09-1.87) | 0.94 (0.73-1.22) |
| China | 1.24 (0.74-2.07) | 1.24 (0.74-2.08) | 0.77 (0.47-1.27) | 1.32 (0.79-2.21) | 1.15 (0.69-1.93) | 0.77 (0.47-1.28) |
| Colombia | 1.69 (1.05-2.73) | 1.68 (1.04-2.72) | 1.15 (0.70-1.89) | 1.52 (0.94-2.44) | 1.59 (0.99-2.57) | 1.07 (0.65-1.75) |
| Croatia | 1.03 (0.57-1.87) | 1.03 (0.57-1.87) | 0.89 (0.49-1.62) | 0.82 (0.45-1.49) | 0.99 (0.55-1.79) | 0.84 (0.46-1.54) |
| Czech Republic | 2.51 (1.38-4.54) | 2.48 (1.37-4.50) | 1.99 (1.08-3.64) | 2.20 (1.21-4.00) | 2.43 (1.34-4.40) | 1.88 (1.02-3.45) |
| Denmark | 0.86 (0.58-1.27) | 0.85 (0.58-1.26) | 0.83 (0.57-1.22) | 0.82 (0.55-1.21) | 0.81 (0.55-1.19) | 0.84 (0.57-1.23) |
| Eritrea | 0.93 (0.63-1.37) | 0.92 (0.62-1.36) | 0.54 (0.37-0.80) | 0.78 (0.53-1.16) | 0.81 (0.55-1.20) | 0.54 (0.37-0.79) |
| Estonia | 0.34 (0.08-1.35) | 0.33 (0.08-1.33) | 0.28 (0.07-1.13) | 0.30 (0.08-1.22) | 0.33 (0.08-1.31) | 0.28 (0.07-1.12) |
| Ethiopia | 1.29 (0.85-1.95) | 1.29 (0.85-1.94) | 0.76 (0.51-1.14) | 1.13 (0.74-1.71) | 1.21 (0.80-1.83) | 0.74 (0.50-1.11) |
| Finland | 1.37 (1.15-1.62) | 1.34 (1.14-1.59) | 0.95 (0.81-1.11) | 1.29 (1.09-1.53) | 1.32 (1.11-1.56) | 0.93 (0.80-1.09) |
| France | 0.74 (0.31-1.76) | 0.74 (0.31-1.75) | 0.79 (0.33-1.87) | 0.71 (0.30-1.66) | 0.72 (0.31-1.72) | 0.76 (0.32-1.79) |
| Germany | 1.26 (0.92-1.74) | 1.25 (0.91-1.72) | 1.20 (0.87-1.64) | 1.22 (0.89-1.67) | 1.23 (0.90-1.70) | 1.18 (0.86-1.62) |
| Greece | 1.68 (1.14-2.48) | 1.68 (1.14-2.48) | 0.98 (0.67-1.44) | 1.39 (0.94-2.05) | 1.58 (1.07-2.33) | 0.94 (0.64-1.38) |
| Hungary | 1.23 (0.75-2.01) | 1.21 (0.74-1.98) | 0.96 (0.59-1.55) | 1.08 (0.66-1.77) | 1.18 (0.72-1.94) | 0.92 (0.57-1.50) |
| India | 1.19 (0.78-1.81) | 1.18 (0.78-1.80) | 0.88 (0.59-1.31) | 1.17 (0.78-1.77) | 1.15 (0.76-1.75) | 0.84 (0.56-1.24) |
| Iran | 1.27 (1.09-1.49) | 1.27 (1.09-1.49) | 0.69 (0.60-0.80) | 0.99 (0.85-1.16) | 1.14 (0.97-1.33) | 0.64 (0.55-0.74) |
| Iraq | 1.29 (1.05-1.57) | 1.29 (1.05-1.57) | 0.87 (0.72-1.06) | 1.13 (0.92-1.38) | 1.21 (0.99-1.47) | 0.80 (0.66-0.97) |
| Italy | 1.26 (0.69-2.28) | 1.26 (0.70-2.29) | 1.14 (0.64-2.03) | 1.17 (0.65-2.13) | 1.21 (0.67-2.19) | 1.11 (0.62-1.98) |
| Lebanon | 1.73 (1.34-2.22) | 1.72 (1.33-2.21) | 0.94 (0.74-1.20) | 1.32 (1.03-1.70) | 1.54 (1.19-1.98) | 0.89 (0.70-1.14) |
| Netherlands | 2.05 (1.23-3.41) | 2.07 (1.24-3.44) | 2.26 (1.38-3.71) | 2.07 (1.24-3.44) | 1.99 (1.20-3.32) | 2.27 (1.39-3.70) |
| North Vietnam | 1.32 (0.78-2.25) | 1.33 (0.78-2.26) | 0.86 (0.51-1.46) | 1.21 (0.71-2.05) | 1.18 (0.70-2.00) | 0.85 (0.50-1.44) |
| Norway | 1.00 (0.69-1.43) | 0.99 (0.69-1.42) | 0.98 (0.68-1.40) | 1.01 (0.70-1.45) | 0.96 (0.67-1.39) | 1.02 (0.71-1.46) |
| Other Africa | 1.48 (1.17-1.86) | 1.47 (1.16-1.85) | 0.84 (0.67-1.05) | 1.24 (0.98-1.56) | 1.36 (1.08-1.72) | 0.79 (0.63-0.99) |
| Other Asia | 1.75 (1.31-2.34) | 1.74 (1.30-2.33) | 0.99 (0.75-1.31) | 1.49 (1.12-1.99) | 1.64 (1.22-2.19) | 0.93 (0.70-1.23) |
| Other EU28 | 0.98 (0.69-1.39) | 0.98 (0.69-1.39) | 0.83 (0.58-1.17) | 0.83 (0.59-1.18) | 0.93 (0.66-1.33) | 0.79 (0.56-1.11) |
| Other Middle East | 1.48 (1.01-2.16) | 1.47 (1.01-2.16) | 0.75 (0.51-1.10) | 1.14 (0.78-1.67) | 1.30 (0.89-1.91) | 0.70 (0.48-1.03) |
| Other non-EU Europe | 1.19 (0.91-1.56) | 1.19 (0.90-1.55) | 0.81 (0.62-1.05) | 0.93 (0.71-1.22) | 1.11 (0.85-1.45) | 0.77 (0.59-1.00) |
| Other Nordics | 1.64 (0.82-3.27) | 1.65 (0.83-3.28) | 1.68 (0.84-3.36) | 1.63 (0.81-3.26) | 1.61 (0.81-3.21) | 1.67 (0.83-3.35) |
| Other North America | 1.13 (0.71-1.79) | 1.13 (0.71-1.80) | 0.85 (0.55-1.34) | 1.03 (0.65-1.64) | 1.07 (0.67-1.71) | 0.83 (0.53-1.30) |
| Other Oceania | 0.81 (0.26-2.53) | 0.80 (0.26-2.50) | 0.86 (0.27-2.74) | 0.78 (0.25-2.45) | 0.79 (0.26-2.47) | 0.82 (0.26-2.62) |
| Other South America | 2.01 (1.40-2.89) | 2.03 (1.41-2.91) | 1.31 (0.92-1.87) | 1.79 (1.25-2.57) | 1.91 (1.33-2.74) | 1.19 (0.83-1.70) |
| Pakistan | 1.34 (0.74-2.41) | 1.33 (0.74-2.40) | 0.73 (0.41-1.30) | 1.12 (0.62-2.02) | 1.19 (0.66-2.15) | 0.68 (0.38-1.22) |
| Peru | 1.39 (0.80-2.40) | 1.40 (0.81-2.42) | 0.76 (0.44-1.32) | 1.24 (0.72-2.15) | 1.33 (0.77-2.30) | 0.69 (0.40-1.20) |
| Philippines | 1.31 (0.82-2.08) | 1.30 (0.81-2.07) | 0.95 (0.60-1.50) | 1.30 (0.82-2.06) | 1.25 (0.79-2.00) | 0.92 (0.58-1.46) |
| Poland | 1.07 (0.84-1.38) | 1.07 (0.84-1.37) | 0.84 (0.66-1.07) | 0.87 (0.68-1.12) | 1.03 (0.80-1.31) | 0.79 (0.62-1.01) |
| Romania | 1.21 (0.84-1.75) | 1.21 (0.84-1.74) | 0.92 (0.64-1.32) | 0.97 (0.68-1.40) | 1.15 (0.80-1.65) | 0.88 (0.61-1.26) |
| Russia | 0.85 (0.51-1.44) | 0.86 (0.51-1.44) | 0.68 (0.40-1.14) | 0.70 (0.42-1.17) | 0.80 (0.48-1.35) | 0.62 (0.37-1.04) |
| Somalia | 0.98 (0.68-1.43) | 0.97 (0.67-1.42) | 0.44 (0.30-0.64) | 0.82 (0.56-1.19) | 0.86 (0.59-1.26) | 0.44 (0.30-0.64) |
| South Vietnam | 2.63 (1.41-4.92) | 2.65 (1.42-4.94) | 1.24 (0.68-2.24) | 2.29 (1.23-4.29) | 2.51 (1.35-4.68) | 1.16 (0.64-2.11) |
| Soviet Union | 1.41 (0.70-2.83) | 1.42 (0.71-2.84) | 1.07 (0.53-2.17) | 1.19 (0.59-2.38) | 1.34 (0.67-2.68) | 0.96 (0.48-1.95) |
| Spain | 0.89 (0.40-1.98) | 0.88 (0.39-1.97) | 0.85 (0.40-1.85) | 0.82 (0.37-1.83) | 0.84 (0.38-1.89) | 0.79 (0.36-1.72) |
| Sri Lanka | 1.93 (1.09-3.43) | 1.93 (1.09-3.42) | 1.23 (0.70-2.15) | 1.80 (1.02-3.19) | 1.84 (1.04-3.26) | 1.19 (0.69-2.08) |
| Syria | 1.31 (1.10-1.55) | 1.30 (1.09-1.54) | 0.72 (0.62-0.85) | 1.00 (0.84-1.19) | 1.09 (0.91-1.29) | 0.69 (0.59-0.81) |
| Thailand | 0.93 (0.63-1.36) | 0.92 (0.62-1.35) | 0.55 (0.38-0.81) | 0.90 (0.61-1.32) | 0.84 (0.57-1.24) | 0.56 (0.39-0.83) |
| Turkey | 1.81 (1.47-2.23) | 1.81 (1.47-2.23) | 0.96 (0.79-1.18) | 1.43 (1.16-1.76) | 1.65 (1.34-2.04) | 0.94 (0.77-1.15) |
| Yugoslavia | 1.83 (1.54-2.16) | 1.81 (1.53-2.14) | 1.12 (0.96-1.31) | 1.47 (1.24-1.74) | 1.73 (1.46-2.04) | 1.08 (0.92-1.26) |

**Table S3:** Incidence rate ratios (IRR) with 95% confidence intervals (95%CI) estimated with Poisson regression with long COVID diagnosis as outcome. Results are for additional adjustments in which wave the infection was confirmed. The selected models are model M4 which adjusts for age, sex, vaccinations, and wave; and model M6 which adjusts for age, sex, vaccinations, disease severity, underlying health, social factors, and wave. Only estimates by country/region of birth and wave are shown, all others available upon request.

|  | **M4 +waves** | **M6 +waves** |
| --- | --- | --- |
| **Country/region of origin** |  |  |
| Sweden | 1(ref) | 1(ref) |
| East Europe | 1.17 (1.06-1.30) | 0.91 (0.83-0.99) |
| Finland | 1.28 (1.08-1.52) | 0.93 (0.80-1.09) |
| Horn of Africa | 0.88 (0.70-1.10) | 0.55 (0.44-0.68) |
| Middle East | 1.05 (0.95-1.16) | 0.69 (0.63-0.76) |
| North America | 1.03 (0.65-1.64) | 0.84 (0.54-1.31) |
| Other Africa | 1.25 (0.99-1.57) | 0.81 (0.65-1.02) |
| Other Asia | 1.27 (1.06-1.51) | 0.78 (0.65-0.92) |
| Other Nordic | 0.97 (0.75-1.25) | 0.97 (0.76-1.24) |
| South America | 1.36 (1.13-1.64) | 0.97 (0.81-1.16) |
| South Asia | 1.13 (0.91-1.40) | 0.71 (0.58-0.87) |
| West Europe | 1.11 (0.91-1.34) | 1.07 (0.89-1.28) |
| **Wave** |  |  |
| Wave 1 | 1(ref) | 1(ref) |
| Wave 2 | 0.67 (0.60-0.74) | 1.57 (1.43-1.72) |
| Wave 3 | 0.42 (0.36-0.49) | 0.99 (0.87-1.13) |

**Table S4:** Incidence rate ratios (IRR) with 95% confidence intervals (95%CI) estimated with Poisson regression with long COVID diagnosis. Results are for additional adjustments for in which wave the infection was confirmed and an interaction between wave and country/region is included in model. The selected models are model M4 which adjusts for age, sex, vaccinations, and wave; and model M6 which adjusts for age, sex, vaccinations, disease severity, underlying health, social factors, and wave. Only estimates by country/region of birth and wave are shown, all others available upon request. IRR are shown for country/region within each wave with residents born in Sweden as reference.

| **M4 + waves + interaction** |  |  |  |
| --- | --- | --- | --- |
|  | Wave 1 | Wave 1 | Wave 3 |
| Sweden | 1(ref) | 1(ref) | 1(ref) |
| East Europe | 1.23 (0.93-1.62) | 1.18 (1.04-1.35) | 1.12 (0.92-1.36) |
| Finland | 1.07 (0.69-1.66) | 1.30 (1.05-1.61) | 1.39 (1.00-1.93) |
| Horn of Africa | 1.12 (0.69-1.83) | 0.96 (0.71-1.28) | 0.53 (0.30-0.94) |
| Middle East | 1.09 (0.84-1.42) | 1.05 (0.92-1.19) | 1.04 (0.84-1.29) |
| North America | 3.07 (1.35-6.96) | 0.78 (0.41-1.50) | 0.75 (0.24-2.33) |
| Other Africa | 1.94 (1.17-3.22) | 1.26 (0.94-1.70) | 0.85 (0.50-1.44) |
| Other Asia | 1.72 (1.08-2.74) | 1.32 (1.07-1.64) | 0.88 (0.57-1.36) |
| Other Nordic | 1.16 (0.57-2.34) | 0.93 (0.68-1.28) | 0.98 (0.59-1.63) |
| South America | 1.75 (1.13-2.72) | 1.36 (1.07-1.72) | 1.12 (0.74-1.72) |
| South Asia | 1.30 (0.72-2.32) | 1.19 (0.92-1.54) | 0.77 (0.42-1.39) |
| West Europe | 1.33 (0.77-2.32) | 1.13 (0.89-1.44) | 0.96 (0.67-1.39) |
| **M6 + waves + interaction** |  |  |  |
|  | Wave 1 | Wave 2 | Wave 3 |
| Sweden | 1(ref) | 1(ref) | 1(ref) |
| East Europe | 0.78 (0.59-1.03) | 0.94 (0.84-1.06) | 1.05 (0.87-1.27) |
| Finland | 0.81 (0.53-1.25) | 0.91 (0.75-1.10) | 1.16 (0.84-1.60) |
| Horn of Africa | 0.47 (0.29-0.77) | 0.68 (0.51-0.89) | 0.49 (0.28-0.86) |
| Middle East | 0.57 (0.44-0.73) | 0.73 (0.66-0.82) | 0.87 (0.71-1.08) |
| North America | 2.21 (1.03-4.75) | 0.66 (0.35-1.25) | 0.63 (0.21-1.89) |
| Other Africa | 0.84 (0.51-1.41) | 0.86 (0.64-1.14) | 0.71 (0.42-1.19) |
| Other Asia | 0.89 (0.56-1.41) | 0.81 (0.66-1.00) | 0.77 (0.50-1.19) |
| Other Nordic | 1.24 (0.61-2.52) | 0.95 (0.70-1.30) | 0.99 (0.59-1.64) |
| South America | 0.95 (0.61-1.48) | 0.95 (0.76-1.18) | 1.16 (0.76-1.76) |
| South Asia | 0.73 (0.41-1.29) | 0.80 (0.63-1.02) | 0.62 (0.35-1.10) |
| West Europe | 1.21 (0.69-2.11) | 1.07 (0.85-1.35) | 1.03 (0.72-1.47) |

**Figure S1:** Predicted counts of a long COVID diagnosis using average marginal effects for the modification effects of sex on the association between region of origin and the outcome.


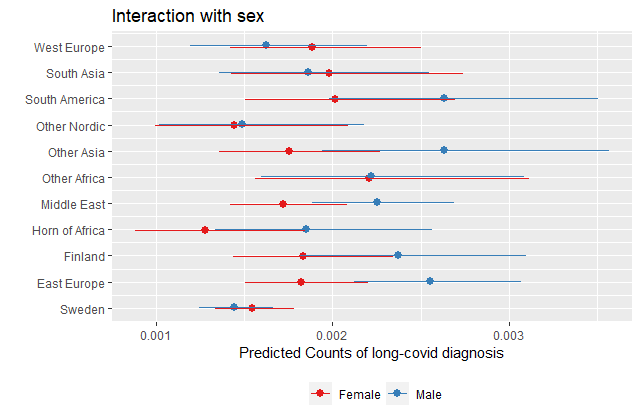


**Figure S2:** Predicted counts of a long COVID diagnosis using average marginal effects for the modification effects of underlying health as measured by Charlson comorbidity index (CCI) on the association between region of origin and the outcome.


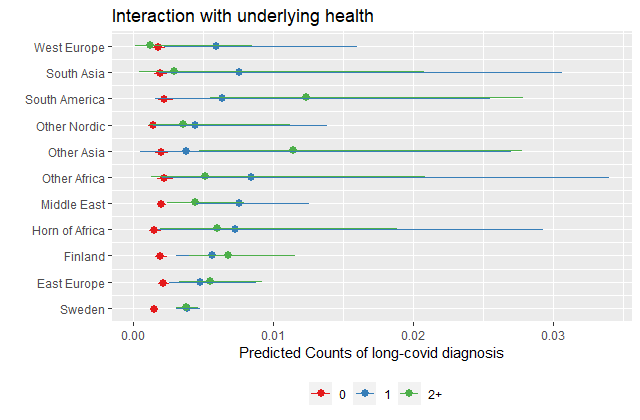


**Figure S3:** Predicted counts of a long COVID diagnosis using average marginal effects for the modification effects of underlying health as measured by disease severity.


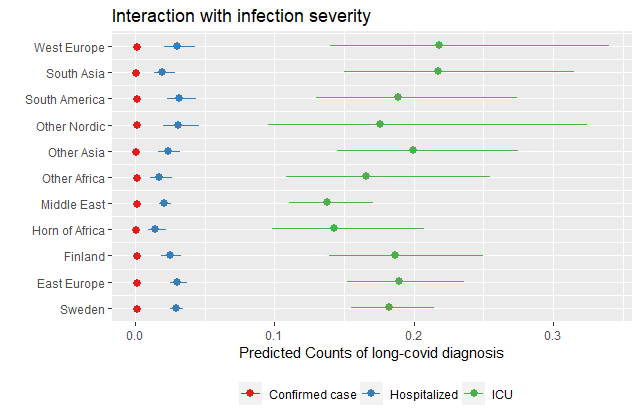


**Figure S4:** Predicted counts of a long COVID diagnosis using average marginal effects for the modification effects of vaccination status.


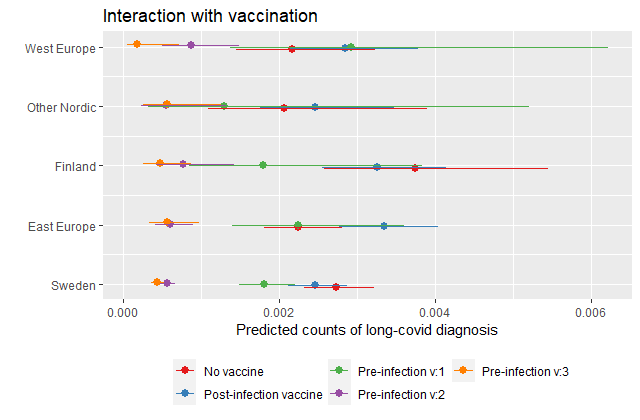


**Figure S5:** Predicted counts of a long COVID diagnosis using average marginal effects for the modification effects of disposable income.


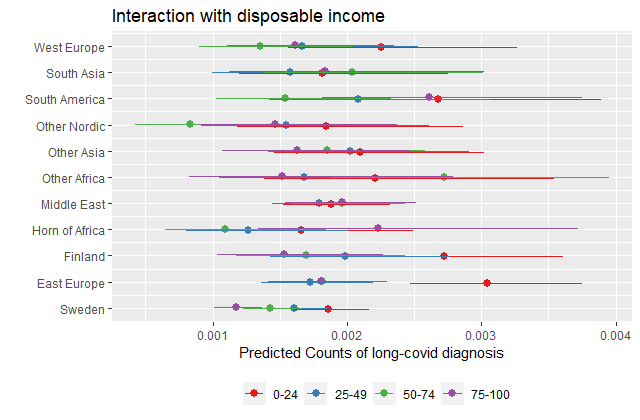


**Figure S6:** Predicted counts of a long COVID diagnosis using average marginal effects for the modification effects of education.


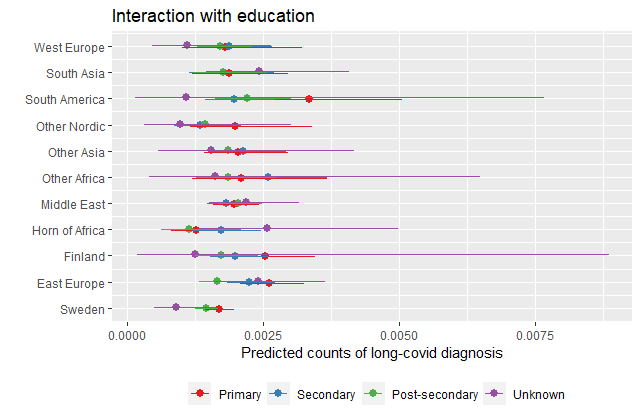


Note: For the supplementary figures some country/region origin groups were excluded due to very large confidence intervals.
